# Orally Administered Phlorotannins from *Eisenia arborea* Suppress Chemical Mediator Release and Cyclooxygenase-2 Signaling to Alleviate Mouse Ear Swelling

**DOI:** 10.3390/md16080267

**Published:** 2018-08-02

**Authors:** Yoshimasa Sugiura, Masakatsu Usui, Hirotaka Katsuzaki, Kunio Imai, Makoto Kakinuma, Hideomi Amano, Masaaki Miyata

**Affiliations:** 1 Laboratory of Food Function and Biochemistry, Department of Food Science and Technology, National Research and Development Agency, Japan Fisheries Research and Education Agency, National Fisheries University, Shimonoseki 759-6595, Japan; usuim@fish-u.ac.jp (M.U.); mmiyata@fish-u.ac.jp (M.M.); 2 Laboratory of Bioorganic Chemistry, Graduate School of Bioresources, Mie University, Tsu 514-8507, Japan; katsuzak@bio.mie-u.ac.jp (H.K.); imai@bio.mie-u.ac.jp (K.I.); 3 Laboratory of Marine Biochemistry, Graduate School of Bioresources, Mie University, Tsu 514-8507, Japan; kakinuma@bio.mie-u.ac.jp (M.K.); amano@bio.mie-u.ac.jp (H.A.)

**Keywords:** brown algae, phlorotannins, anti-allergy, mouse ear swelling, arachidonic acid cascade, chemical mediators

## Abstract

Phlorotannin is the collective term for polyphenols derived from brown algae belonging to the genera *Ascopyllum*, *Ecklonia*, *Eisenia*, *Fucus* and *Sargassum* etc. Since the incidence of allergies is currently increasing in the world, there is a focus on phlorotannins having anti-allergic and anti-inflammatory effects. In this study, six purified phlorotannins (eckol; 6,6′-bieckol; 6,8′-bieckol; 8,8′-bieckol; phlorofucofuroeckol (PFF)-A and PFF-B) from *Eisenia arborea*, orally administered to mice, were examined for their suppression effects on ear swelling. In considering the suppression, we also examined whether the phlorotannins suppressed release of chemical mediators (histamine, leukotriene B_4_ and prostaglandin E_2_), and mRNA expression and/or the activity of cyclooxygenase-2 (COX-2), using RBL-2H3 cells, a cultured mast cell model. Results showed that the phlorotnannins exhibited suppression effects in all experiments, with 6,8′-bieckol, 8,8′-bieckol and PFF-A showing the strongest of these effects. In conclusion, orally administered phlorotannins suppress mouse ear swelling, and this mechanism apparently involves suppression of chemical mediator release and COX-2 mRNA expression or activity. This is the first report of the anti-allergic effects of the orally administered purified phlorotannins in vivo. Phlorotannins show potential for use in functional foods or drugs.

## 1. Introduction

In developed countries, an increase in the incidence of allergies such as food allergy, asthma, atopic dermatitis and hay fever, has become an acute problem [1]. In particular, according to the Health, Labour and Welfare Ministry of Japan, half of the Japanese population may suffer allergies of some kind [2]. In Japan, where allergies are a societal problem, natural remedies and foods possessing anti-allergic and anti-inflammatory effects are needed, and much research into compounds and products with anti-allergic effects is underway. Phenolic compounds (polyphenols) are a well-known class of natural anti-allergic natural compounds that are derived from food materials. Polyphenols in terrestrial plants include tea catechins, flavonoids and tannins. In particular, the anti-allergenic mechanisms of tea catechins are well reported [3].

**Figure 1:**
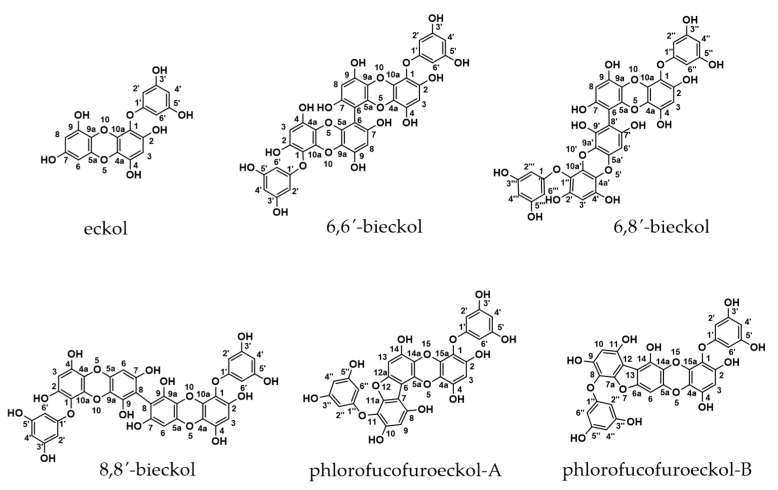
The chemical structures of six phlorotannins.

Polyphenols are also present in seaweed and the collective term for seaweed polyphenol is phlorotannins. Phlorotannins, such as eckol, dieckol, 6,6′-bieckol, 8,8′-bieckol and phlorofucofuroeckol (PFF)-A, are well-known components of brown algae [4]. They are secondary metabolites of photosynthesis and protect algae from ultraviolet (UV) radiation and from consumption by herbivores. In humans, phlorotannins incorporated into health foods or used as drugs are useful physiologically-active compounds that can be used to prevent lifestyle-related diseases, such as cancer and diabetes [5]. There are, therefore, several reports of their anti-allergenic and anti-inflammatory effects. In recent reports, phlorotannin extracts from Fucales inhibited lipoxygenase activity and nitrite oxide production from RAW 264.7 cells, and some phlorotannins from *Ecklonia cava*, *Ecklonia stolonifera* and *Eisenia bicyclis* suppressed inflammation through the MAPK cascade or through chemical mediator production in BV-2, HaCaT and RAW 264.7 cells [6,7,8,9,10]. In our previous study, we isolated six phlorotannins (eckol; 6,6′-bieckol; 6,8′-bieckol; 8,8′-bieckol; PFF-A and PFF-B, Figure 1) from the brown alga *Eisenia arborea* [11,12], and reported the anti-allergenic and anti-inflammatory effects of their phlorotannins [13,14]. Validation studies of effective extraction or purification methods of them using supercritical fluid extraction, pressurized liquid extraction, liquid–liquid or solid–liquid separation, chromatographic methods and liquid chromatography high-resolution mass spectrometry have also been reported since publication of the usefulness of phlorotannins [15,16,17,18].

While the anti-allergenic effects of terrestrial plant polyphenols are well documented, there are fewer published studies on phlorotannins on the anti-allergenic and anti-inflammatory effects of phlorotannins and most reports concern in vitro studies. In this context, we aimed to the efficacy of phlorotannins as anti-allergenic and anti-inflammatory compounds in vivo. In a previous study, we had investigated the inhibitory effects of six pirified phlorotannins on allergic inflammation-related enzymatic activities [13,14] and their efficacy in suppressing mouse ear swelling [19,20]. When percutaneously administered, the phlorotannins suppressed the mouse ear swelling by sensitizers including arachidonic acid (AA), 12-*O*-tetradecanoylphorbol-13-acetate (TPA) and oxazolone (OXA) [19,20]. However, the therapeutic effects of phlorotannins remains to be investigated. In addition, the efficacy of phlorotanins when given in food (intestinal absorption) or when taken as an anti-allergenic drug needs to be investigated. In fact, fucoxanthin, a marine carotenoid, and isoflavonoids possess bioactive properties to convert to fucoxanthinol and equol, respectively, in the intestine [21,22]. Therefore, in this study, we also examined whether the six purified phlorotannins were administered orally suppressed mouse ear swelling that was induced by AA, TPA and OXA in vivo. Moreover, the ear swelling induced by the three sensitizers we used involves cyclooxygenase (COX)-2 expression or activity, which is attributed to chemical mediator release such as histamine, leukotrienes and prostaglandins [23,24,25]. Accordingly, to investigate the suppression mechanism in vitro, we examined the effects of the six phlorotannins on chemical mediator release and COX-2 expression or activity in a cultured mast cell line, rat basophile leukemia (RBL)-2H3.

## 2. Results

### 2.1. Suppression of Mouse Ear Swelling

To investigate the anti-inflammatory effects of the six purified phlorotannins in vivo, we induced ear swelling in Institute of Cancer Research (ICR) mice using three sensitizers (AA, TPA and OXA), and we administered orally the purified phlorotannins. In parallel, we used terrestrial plant polyphenol epigallocatechin gallate (EGCG) well-known for its anti-allergenic and anti-inflammatory properties [3].

As shown in Table 1, the six phlorotannins suppressed AA-induced mouse ear swelling. Ear swelling values for 6,6′-bieckol, 6,8′-bieckol, PFF-A and PFF-B were significantly (*p* < 0.05) lower than they were for controls. The suppression ratios for 6,6′-bieckol, 6,8′-bieckol and PFF-B tended to be higher than they were for epigallocatechin gallate (EGCG). The six phlorotannins also suppressed TPA-induced mouse ear swelling. Ear swelling values for the six phlorotannins were significantly (*p* < 0.05) lower than controls. The suppression ratio for 6,8′-bieckol was significantly (*p* < 0.05) higher, and the ratios for eckol and PFF-B tended to be higher than for EGCG. Moreover, the six phlorotannins suppressed OXA-induced mouse ear swelling. Ear swelling values for 6,8′-bieckol, 8,8′-bieckol and PFF-B were significantly (*p* < 0.05) lower than for those of controls. The suppression ratios for 6,8′-bieckol and PFF-B were significantly (*p* < 0.05) higher, and the ratio of 8,8′-bieckol tended to be higher than for EGCG. In spite of the weakest suppression by EGCG at 75 nmol (5.7%), 6,8′-bieckol at 75 nmol exhibited the strongest suppression (77.8%). EGCG at 200 nmol suppressed ear swelling by 34.3% (data not shown).

The apparent suppression ratios of the six purified phlorotannins except for eckol in AA sensitization were higher than those of EGCG, in each sensitizer experiment. Difference of suppression effects of eckol or 6,6′-bieckol on each ear swelling induced by the three sensitizers was observed. Using any of the three sensitizers, 8,8′-bieckol and PFF-A exhibited a reasonable suppression ratio in the range of 21.0%–32.3%. However, 6,8′-bieckol and PFF-B clearly suppressed the ear swelling induced by all three sensitizers, suggesting that these two phlorotannins have potency as anti-inflammatory compounds.

### 2.2. Suppression of Chemical Mediator Release in RBL-2H3 Cells

To investigate the mouse ear swelling suppression mechanism conveyed by the six purified phlorotannins, we tested the potential degranulation or chemical mediator release in RBL cultured mast cells. The RBL cells were stimulated by antigen-antibody reaction or A23187. According to previously reported phlorotannin concentration ranging 1 to 200 μM in cultured model cell experiments [7,8,10,26,27,28,29], we chose a concentration range from 10 to 200 μM in our experiments.

#### 2.2.1. Anti-Degranulation in RBL Cells Stimulated by Antigen-Antibody or A23187

As shown in Table 2, at concentrations of either 10 or 100 μM, 8,8′-bieckol and PFF-A significantly suppressed degranulation in RBL cells (*p* < 0.01), at a better efficacy than EGCG (in fact, no degranulation in RBL cells treated with these two phlorotannins at 100 μM was observed). At 100 μM, eckol and 6,8′-bieckol suppressed degranulation in RBL cells, compared with EGCG. At 1 μM, although 8,8′-bieckol did not suppress degranulation, the suppression ratio for PFF-A was 13.9% (data not shown). In RBL cells stimulated by A23187, 8,8′-bieckol suppressed degranulation in RBL cells significantly (*p* < 0.01) more than in EGCG at 10 μM concentrations. No suppression by eckol, 6,6′-bieckol, PFF-A and PFF-B was observed. At 100 μM, suppression by PFF-A tended to be higher than for EGCG, and suppression of 8,8′-bieckol was comparable to that of EGCG.

The apparent suppression ratios of the six purified phlorotannins at 100 μM were lower when stimulation was performed by A23187 than by the anti-antibody, a difference particularly striking in the case if eckol.

#### 2.2.2. Suppression of Chemical Mediator Release from RBL Cells

As shown in Table 3, five phlorotannins (eckol; 6,6′-bieckol; 6,8′-bieckol; 8,8′-bieckol and PFF-A) and EGCG at 100 μM suppressed histamine release. The effect of PFF-B was weaker than that of these five phlorotannins, showing a suppressive ratio at 200 μM of 37.1%. Thus, with respect to the suppression of histamine release, the effects of phlorotannins (other than PFF-B) appear comparable to or significantly stronger than for EGCG. The suppression effects of phlorotannins (except for 6,6′-bieckol) on leukotriene B_4_ release were comparable to EGCG. At 200 μM, a suppression effect for 6,6′-bieckol was observed. The phlorotannins and EGCG at concentrations of both 10 and 100 μM also exhibited suppression effects on prostaglandin E_2_ release. At 100 μM, the suppression ratio of PFF-A was comparable to that of EGCG.

In leukotriene B_4_ and prostaglandin E_2_ release, the apparent effect of 6,6′-bieckol was weakest compared with the other five phlorotannins and EGCG. In histamine release, that of PFF-B was weakest. At 100 μM, eckol, 8,8′-bieckol and PFF-A exhibited definite suppression with ratios over 60%.

### 2.3. Inhibition of Cyclooxygenase-2 (COX-2) mRNA Expression and COX-2 Activity

The ear swelling induced by the three sensitizers (AA, TPA and OXA) closely involves an increase in cyclooxygenase (COX)-2 expression or activity [23,24,25]. In this study, we therefore investigated inhibitory effects of phlorotannins on each step: COX-2 mRNA expression and COX-2 enzymatic activity. Since the suppression effect of phlorotannins on COX-2 mRNA expression was examined using RBL cells, the concentration of the phlorotannins were 10 μM and 100 μM. On the contrary, since COX-2 reagent and not RBL cells were used in the experiment for inhibitory effects of the phlorotannins on COX-2 activity, it was conducted at the higher concentration.

**Figure 2:**
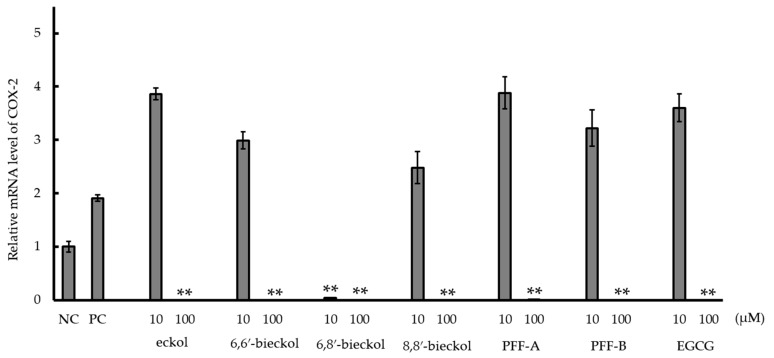
The suppression effect of phlorotannins on COX-2 mRNA expression in RBL cells stimulated by A23187. Values were calculated from the results of triplicate experiments and are expressed as mean ± SD. Reproducibility was confirmed by repeated runs. Asterisks indicate that mRNA level of the test group was significantly lower than mRNA level of PC (** *p* < 0.01). NC, negative control without A23187; PC, positive control with A23187; PFF, phlorofucofuroeckol; and EGCG, epigallocatechin gallate.

At the concentration of 10 μM, only 6,8′-bieckol significantly (*p* < 0.01) inhibited COX-2 mRNA expression in RBL cells, but at 100 μM all six phlorotannins and EGCG showed significant inhibition effects (Figure 2). 

**Figure 3:**
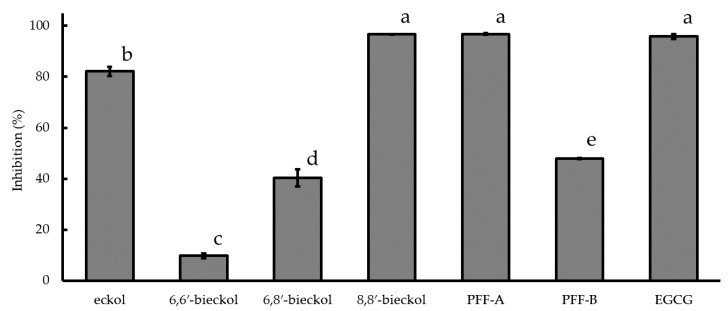
The inhibitory effects of phlorotannins at 500 µM on COX-2 activity. Values were calculated from the results of triplicate experiments and are expressed as mean ± SD. Reproducibility was confirmed by repeated runs. Differences between groups indicated by different letters are statistically significant (*p* < 0.01). PFF, phlorofucofuroeckol; and EGCG, epigallocatechin gallate.

Since no inhibition effects on COX-2 activity was observed at 400 μM with 6,6′-bieckol and 6,8′-bieckol [14], the phlorotannins were examined at a concentration of 500 μM. As shown in Figure 3, 6,6′-bieckol and 6,8′-bieckol also inhibited COX-2 activity, with inhibition ratios of 9.8% and 40.4%, respectively, while inhibition with 8,8′-bieckol (96.6%) and PFF-A (96.8%) was comparable to that of EGCG (95.8%). The inhibition ratios of eckol and PFF-B were 82.1% and 47.9%, respectively, and were significantly lower than that of EGCG.

## 3. Discussion

This is the first reported account that orally administered purified phlorotannins (eckol; 6,6′-bieckol; 6,8′-bieckol; 8,8′-bieckol; phlorofucofuroeckol (PFF)-A and PFF-B) suppress mouse ear swelling induced by the three sensitizers AA, TPA and OXA. In our previous study, these percutaneously administered phlorotannins also suppressed ear swelling [19,20]. In the case of percutaneous administration, the phlorotannins directly influence the ear swelling reaction in tissues. However, when considering phlorotannins as health food ingredients, experiments involving oral administration are necessary to demonstrate whether the phlorotannins are absorbed from the intestinal tract to exert their suppression effects and this was shown to be the case in our study (Table 1). Results from a clinical trial using phlorotannin extract of a brown alga, *Ascophyllum nodosum*, reported that metabolites of phlorotannins composed of phloroglucinol oligomer were found in urine and plasma [30]. Therefore, it might be the metabolites of the phlorotannins that suppressed ear swelling. On the other hand, the inflammation mechanisms of the three sensitizers are divided into two types; immediate-acting (AA or TPA) and a delayed allergic reaction (OXA). The inflammation caused by AA and TPA follows topical application, while the reaction caused by OXA is systemic via immune reactions [25,31]. Whether the suppression effects of the phlorotannins on inflammation seen here can be attributed to a topical effect (such as suppression of chemical mediator release) or, in the case of OXA-induced inflammation to an effect regulating the immune system is unclear. Regarding orally administered terrestrial plant polyphenols, tea theaflavins decreased levels of IL-12 and IFN-γ in mouse ear swelling induced by OXA, and isoflavones suppressed IFN-γ and CCL24 mRNA expression and OXA-specific IgG levels in mice with delayed-type hypersensitivity response induced by OXA or 2,4-dinitrofluorobenzene [32,33,34]. Therefore, phlorotannins might also modulate the immune response following OXA-induced ear swelling, regulating levels of antibodies and cytokines. With respect to the suppression effects of phlorotannins on delayed-type hypersensitivity response induced by OXA, further studies using ICR mice are required. Thus, the novel fact that the six orally administered purified phlorotannins suppressed ear swelling in this study indicates their potential to be active compounds of health foods.

Inflammation caused by AA, TPA and OXA is associated with cyclooxygenase (COX)-2 expression or activity, and attributed to the release of chemical mediator such as histamine, leukotrienes and prostaglandins [23,24,25]. Mast cells aggregate in inflamed tissue and are involved in inflammatory reactions [35]. Therefore, we investigated the mechanism of the suppression effects of the six phlorotannins on mouse ear swelling using a rat mast cell line (RBL-2H3 cells). Using enzyme-linked immunosorbent assay (ELISA) or polymerase chain reaction (PCR), we evaluated the suppression of phlorotnnins on COX-2 expression, COX-2 activity and chemical mediator release. In a previous study, phlorotannin extracts at concentration of 1 mg/mL, including the six purified phlorotannins from *Eisenia arborea*, strongly suppressed chemical mediator release [14]. In a novel discovery, the suppression effect of the six purified phlorotannins was confirmed (Table 2 and Table 3) in this study. The suppression effects could be attributed to a potentiation involving both the suppression of chemical mediator release and the inhibition of enzymatic activity (phospholipase A_2_ [PLA_2_], lipoxygenase [LOX] and COX-2) involved in eicosanoid synthesis [14]. In particular, COX-2 expression and activity is critical for inflammation induced by the three sensitizers [23,24,25]. The six purified phlorotannins at 100 μM suppressed COX-2 mRNA expression in RBL cells. In particular, only 6,8′-bieckol at 10 μM was able to suppress COX-2 mRNA expression (Figure 2). Although the suppression effects of purified phlorotannins on COX-2 mRNA expression in the mouse macrophage cell line RAW 264.7 were already demonstrated in a previous study [8,26,36], this is the first report in mast cell line RBL-2H3. The six phlorotannins at 500 μM inhibited COX-2 enzymatic activity (Figure 3). There are reports that purified phlorotannins reduce COX-2 signaling via suppression of MAPK/NF-κB signaling [8,26,27]. Accordingly, the suppression effects of the six purified phlorotannins tested here could be attributed to COX-2 mRNA expression via suppression of MAPK/NF-κB signaling and inhibition of COX-2 activity in mast cells accumulated in inflamed tissues. In this study, although orally administered 6,6′-bieckol and 6,8′-bieckol suppressed ear swelling (Table 1), at 500 μM they inhibited COX-2 activity (Figure 3) less than they inhibited the other four phlorotannins. The same two phlorotannins at 100 μM suppressed COX-2 mRNA expression in RBL cells, compared with the other four phlorotannins (Figure 2). It has been reported that the anti-inflammatory effect of 6,6′-bieckol is via the inhibition of COX-2 expression and activity through suppression of NF-κB and ERK 1/2 signaling [28,29]. The suppression effect of 6,6′-bieckol on ear swelling could be attributable to greater suppression of COX-2 mRNA expression than to inhibition of COX-2 activity. On the other hand, percutaneously administered 6,8′-bieckol tended to suppress ear swelling more strongly than did 6,6′-bieckol [20], and when orally administered it strongly suppressed the ear swelling induced by TPA and OXA (Table 2 and Table 3). The reason would be that the inhibitory effect of 6,8′-bieckol on COX-2 activity at 500 μM and on mRNA expression at 10 μM is significantly stronger than the inhibitory effects of 6,6-bieckol (Figure 2 and Figure 3). As with 6,6′-bieckol, suppression by 6,8′-bieckol of NF-κB and ERK1/2 signaling in COX-2 signaling might be strengthened. The significant suppression by 6,8′-bieckol of COX-2 mRNA expression is a first report.

There is likely to be a relationship between degranulation (Table 2) and chemical mediator release (Table 3). The suppression effects of eckol, 8,8′-bieckol and PFF-A on degranulation or on the chemical mediator release of each were significant or comparable to the suppression effects of EGCG. The suppression effects of 8,8′-bieckol and PFF-A on degranulation stimulated by A23187 were also comparable to those of EGCG (Table 2). The moderate effect of 6,8′-bieckol is also likely to possess such a relationship. The location of hydroxyl groups in polyphenols is critical for anti-allergenic and inflammatory effects [37,38]. The three bieckols used here are dimers of eckol, the bieckols are isomers, and PFF-A and PFF-B are isomers. Among the phlorotannins, the locations of hydroxyl groups and of the electro-organic interaction are different. Considering their structures and the data presented in Figure 2, Table 2 and Table 3, the location of the C-4-OH or C-4′-OH in eckol and the bieckols might be critical for the suppression effect on degranulation and COX-2 mRNA expression. Regarding the relationship between PFF-A and PFF-B, the C-8-OH of PFF-A and the C-11-OH of PFF-B might be critical for suppression of degranulation and chemical mediator release. It could be that, in PFF-B, the interaction between C-11-OH and C-14-OH reduced the suppression effects compared with PFF-A (Table 2 and Table 3). In addition, the geometry of the aromatic ring and main configuration of the eckol skeleton might be critical for the suppression properties of the six phlorotannins. The geometry and configuration could be dictated by interaction among hydroxyl groups in the phlorotannin molecule, hydroxyl bonding between molecules and π–π bonding among the aromatic rings of the molecules. Since some covalent bonds exist between aromatic rings in the dioxin skeleton, biphenyl bond and phenolic ether bond, the 3-dimensional structures of the phlorotannins are rigid and stable. Therefore, the six purified phlorotannins sould exhibit the anti-allergenic and anti-inflammatory effects. To explain a relationship between the structural variation of the phlorotannins and the differences in suppression effects seen in this study, a further demonstration of interactions between stable structures such as phlorotannins and functional proteins involved in inflammatory reactions would be needed.

In this study, the mechanisms of the suppression effects on mouse ear swelling induced by AA, TPA and OXA of the six orally administered purified phlorotannins are partially demonstrated in experiments with RBL cells and in the evaluation for COX-2 signaling. However, since there is not always a relationship between the in vivo data (Table 1) and the in vitro data (Table 2 and Table 3; Figure 2 and Figure 3), evaluations of intestinal absorption and metabolism of the phlorotannins are needed. Moreover, since an immunomodulating effect of phlorotannin extract on mouse ear swelling induced by TPA has been reported [39], measurement of antibodies and cytokines is needed in mice for which phlorotannins are orally administered.

## 4. Materials and Methods

### 4.1. Materials

*E. arborea* samples were collected from the coast of Mugizaki, Mie Prefecture, Japan. Harvested algae were dried and ground into powder according to the procedure outlined by Sugiura et al. [40]. Previously, six phlorotannins (eckol; 6,6′-bieckol; 6,8′-bieckol; 8,8′-bieckol; phlorofucofuroeckol (PFF)-A and PFF-B) (Figure 1) were isolated from powdered algae via methanol/chloroform (M/C) extraction and high performance liquid chromatography (HPLC) (Develosil ODS-5, Nomura Chemical Co., Ltd., Seto, Aichi, Japan) purification [14]. The samples were stored at −80 °C in methanol. Owing to the length of the storage period, we conducted HPLC analysis and 1,1-diphenyl-2-picrylhydrazyl radical scavenging assay [13] to check for phlorotannin decomposition; no decomposition was observed (data not shown). Safety and the absence of toxicity of phlorotannins derived from *Ecklonia cava* were demonstrated [41,42]. And, in our previous study, we observed no diarrhea or abnormal symptoms in any of the experimental animals fed on the dried powder of *E. arborea* [40]. Therefore, we considered that the six purified phlorotannins were adequate as material in functional foods and drugs. For a typical natural inhibitor, epigallocatechin gallate (EGCG; Sigma-Aldrich, St. Louis, MO, USA), well-known for exhibiting anti-allergenic and anti-inflammatory properties [3], was used.

### 4.2. Animals

The ICR strain mouse as a carcinoma model was established by Hauschka et al. [43], and is usually used for evaluating inflammation in primarily immunology, toxicology and pharmacology studies [44]. Accordingly, we used ICR mice as an ear swelling model. The mice (male, four weeks old) were purchased from KBT Oriental Co., Ltd. (Tosu, Saga, Japan) and housed in individual cages kept at 23–26 °C under a 12 h light/dark cycle until testing. A solid AIN-93G diet (KBT Oriental) and tap water were freely available. Throughout the experimental periods, the mice maintained a good appetite. Among all groups, no diarrhea or abnormal symptoms were observed. Among the mice tested, no significant difference of food intake or body weight gain was observed. All of the animal experiments were conducted after receiving permission from the Committee for Use and Care of Laboratory Animals of the National Fisheries University, and in compliance with the Guidelines for Animal Experiments in Research Institutes under the Jurisdiction of the Ministry of Agriculture, Forestry and Fisheries (Approval number, 17-8; 31 March, 2017). 

### 4.3. Anti-Inflammatory Effects on Mouse Ear Swelling

#### 4.3.1. Arachidonic Acid (AA)

AA-induced ear swelling was performed according to the method described by Young et al [45], with some modifications. At 18 h after oral administration of the phlorotannins (75 nmol) dissolved in 400 μL of 10% Tween 60 solution using a stainless feeding needle, 10 μL of AA (12.5 mg/mL in acetone, stored at −20 °C until use; Wako Pure Chemical Industries, Ltd., Osaka, Japan) was spread on the mouse ear. After 1 h, the extent of ear swelling was determined using a thickness gage (547 series, Mitsutoyo Corporation, Kawasaki, Kanagawa, Japan). The relative ratio of the ear swelling suppression was calculated according to the following formula:

(1) $$\;\mathrm{Suppression}\;\mathrm{ratio}\;\left(\%\right)\;=\;\left[1\;-\frac{\left(T\;-T_{0}\right)}{\left(C\;-C_{0}\right)}\right]\times \;100\;$$

where C_0_ is the ear thickness without the test sample before spreading AA, C is the ear thickness without the test sample after spreading AA, T_0_ is the ear thickness with the test sample before spreading AA and T is the ear thickness with the test sample after spreading AA.

#### 4.3.2. 12-*O*-Tetradecanoylphorbol-13-Acetate (TPA)

TPA-induced ear swelling was conducted according to Young et al. [45], with some modifications. The test sample (400 μL) was orally administered at 3 h and 21 h prior to spreading TPA (10 μL) on the mouse’s ear. Immediately before use, a stock solution of TPA (800 μg/mL in acetone, stored at −20 °C until use; Wako) was diluted to 80 μg/mL with acetone. At 4 h after spreading the TPA, the extent of ear swelling was determined using a thickness gage. The relative ratio of the ear swelling suppression was calculated as described for AA above.

#### 4.3.3. Oxazolone (OXA)

OXA-induced ear swelling was performed as described by Suzuki et al. [46], with some modifications. The OXA solution (Sigma-Aldrich) was stored at −20 °C until use. First, 400 μL of the test sample was administered orally at 1 h before spreading 1% OXA dissolved in ethanol (50 μL) on the clean-shaven abdominal region of the mouse. After five days, 400 μL of the test sample was orally administered once again at 1 h before the spreading of 0.5% OXA dissolved in acetone (10 μL) on the mouse’s ear. At 24 h after spreading the OXA, the extent of ear swelling was determined using a thickness gage. The relative ratio of the ear swelling suppression was calculated as described for AA above.

### 4.4. Suppression Effect on Chemical Mediator Release

#### 4.4.1. Cell Culture

RBL-2H3 cells provided by the Health Science Research Resources Bank (JCRB0023, Tokyo, Japan) were cultured in Eagle’s Modified Essential Medium (EMEM, Sigma-Aldrich) supplemented with fetal bovine serum (FBS) (10%; Lot, AAJ208538; HyClone Laboratories, Inc., South-Logan, UT, USA), penicillin (10^5^ U/L; Wako) and streptomycin (100 mg/L; Wako). The cells were transferred every three or four days by trypsinization until they attained confluence.

#### 4.4.2. Cell Stimulation and Anti-Degranulation Assay

The amount of degranulation by the mast cells is known to be proportional to the activity of β-hexosaminidase (β-Hex) [47], therefore the anti-degranulation assay was conducted using stimulated RBL cells based on measurement of the activity of the released β-Hex. RBL cells (2 × 10^5^ cells/well) were precultured and sensitized overnight with 0.2 μg of anti-DNP IgE (Sigma-Aldrich) in a 24-well plate. After removing the anti-DNP IgE, the test samples were added and the cells were incubated for 10 min. Subsequently, the cells were stimulated with 4 μg of DNP-BSA (LSL Co., Ltd., Tokyo, Japan) for 30 min. In the case of stimulation by the calcium ionophore A23187 (Sigma-Aldrich), the precultured cells were treated with the stimulant (1 μM) dissolved in dimethyl sulfoxide (DMSO) for 30 min without the antigen-antibody reaction. The stimulatory reaction was stopped by cooling the reaction vessel on ice for 10 min. The culture medium was divided into three wells of a 96-well plate, and the substrate solution (1 mM *p*-nitrophenyl-*N*-acetyl-β-d-glucosaminide, Wako) was added to each well. After incubation for 60 min at 37 °C, 100 mM sodium bicarbonate was added to each solution to alkalize them and develop a yellow color. The developed color was measured at an optical density of 405 nm to determine the amount of *p*-nitrophenol freed from the substrate by the action of the released enzyme using a microplate reader (Infinite F50, TECAN Austria GmbH, Grodig, Austria). The inhibition ratio of the β-Hex was calculated using the following equation.

(2) $$\;\mathrm{Inhibition}\;\mathrm{ratio}\;\left(\%\right)\;=\;\left[1\;-\;\frac{\left(T\;-\;B\right)}{\left(C\;-\;B\right)}\right]\;\times \;100\;$$

where B is the OD_405_ of the blank cell medium with neither the test sample nor stimulant, C is the OD_405_ of the cell medium without the test sample but with the stimulant, and T is the OD_405_ of the cell medium with both test sample and stimulant.

The potential induction of cell death by the phlorotannins was tested for by Trypan blue exclusion. In the phlorotannin-treated cells, cell death was not observed (data not shown). Moreover, inhibition of the activity of β-Hex in the test samples was not observed.

#### 4.4.3. Determination of Released Chemical Mediators

ELISA methods were employed for determining the amount of chemical mediator released from RBL cells stimulated by the antigen-antibody reaction, as described above, using kits for leukotriene B_4_ and prostaglandin E_2_ (Cayman Chemical Co., Ann Arbor, MI, USA), and histamine (SPI-BIO, Montigny Le Bretonneux, France), according to the protocols recommended by the manufacturers.

### 4.5. Suppression of COX-2 Gene Expression and Enzymatic Activity

#### 4.5.1. Cell Stimulation

The RBL cells were stimulated with A23187, according to the method of Matsui et al. [48], with some modifications. The RBL cells suspended in EMEM were inoculated in each well of a 24-well plate (2 × 10^5^ cells/well) and precultured overnight. The cells were exposed to phlorotannins for 30 min and then stimulated with A23187 (2 μM). After being cultured for 3 h, the cell suspension was collected with a cell scraper.

#### 4.5.2. Quantitative Real-Time Polymerase Chain Reaction (qPCR)

Total RNA was isolated from RBL cells with ISOGEN reagent (Nippon Gene Co. Ltd., Tokyo, Japan), according to the manufacturer’s instructions, and quantified by spectrophotometry at 260 nm. Single-strand cDNA was synthesized using an oligo (dT) primer and High-Capacity cDNA Reverse Transcription Kit (Applied Biosystems, Foster City, CA, USA). The cDNA was used for real-time quantitative polymerase chain reaction (qPCR) using SYBR Premix Ex TaqTM II (Tli RNaseH Plus) (Takara Bio, Inc., Otsu, Shiga, Japan) with the TP870 Thermal Cycler Dice Real Time System (Takara Bio). Relative mRNA levels were calculated by the comparative threshold cycle method. The following specific forward and reverse primers were used for real-time qPCR: COX-2, sense, 5′-CCC ATG TCA AAA CCG TGG TG-3′ and antisense, 5′-CTG TGT TTG GGG TGG GCT TC-3′; and GAPDH, sense, 5′-TGT GTC CGT CGT GGA TCT GA-3′ and antisense, 5′- CCT GCT TCA CCA CCT TCT TGA T-3′.

#### 4.5.3. Inhibitory Effect on COX-2 Activity

Assays for the inhibitory effects of phlorotannins on COX-2 activity as well as ELISA for prostaglandin E_2_ produced from COX-2 enzymatic reaction were conducted using a kit for cyclooxygenase inhibitor screening (Cayman Chemical). The inhibitory effects assay and the ELISA for prostaglandin E_2_ were conducted according to the protocols recommended by the manufacturers.

### 4.6. Statistical Analysis

Data are expressed as mean ± standard deviation (SD). Multiple comparisons between groups were performed with Dunnett’s test using the software Excel Statistics 2016 (Social Survey Research Information Co., Ltd., Tokyo, Japan), with *p* < 0.05 considered statistically significant. In the study of COX-2 activity inhibition, multiple comparisons between groups were performed with the Tukey–Kramer test with the same software, with *p* < 0.05 considered statistically significant.

## 5. Conclusions

In this study, the suppression effect of six phlorotannins (eckol; 6,6′-bieckol; 6,8′-bieckol; 8,8′-bieckol; PFF-A and PFF-B) isolated from *Eisenia arborea* on allergic inflammation were investigated. Using ICR mice sensitized by AA, TPA and OXA as an allergic inflammatory model, the orally administered phlorotannins suppressed ear swelling. To investigate the mechanism of the suppression effect, experiments using a rat mast cell line (RBL-2H3 cells) were performed, and the inhibition effect on COX-2 activity was evaluated with ELISA. Results showed that the phlorotannins suppressed degranulation, the release of chemical mediators (histamine, leukotriene B_4_ and prostaglandin E_2_) and COX-2 mRNA expression, and inhibited COX-2 activity at a concentration of 500 μm. In particular, 6,8′-bieckol exhibited significant suppression on mouse ear swelling and COX-2 mRNA expression, and this result is a first report. In conclusion, these phlorotannins alleviate allergic inflammation via suppression or inhibition of degranulation, release of chemical mediators and COX-2 activation by inflammatory lymphocytes.

## Figures and Tables

**Table 1:** The suppression effects of phlorotannins on sensitizer-induced mouse ear swelling.

**Sensitizer**	**Test Groups**	**Control**	**Eckol**	**6,6′-Bieckol**	**6,8′-Bieckol**
AA	Ear swelling (mm)	0.193 ± 0.026	0.169 ± 0.011	0.112 ** ± 0.049	0.116 ** ± 0.036
	Suppression (%)	—	12.7 ± 5.4	41.9 ^#^ ± 25.4	39.8 ^#^ ± 18.7
TPA	Ear swelling (mm)	0.194 ± 0.038	0.116 ** ± 0.009	0.174 * ± 0.016	0.098 ** ± 0.032
	Suppression (%)	—	40.0 ^#^ ± 4.5	34.2 ± 25.2	49.4 * ± 16.4
OXA	Ear swelling (mm)	0.223 ± 0.024	0.180 ± 0.020	0.183 ± 0.030	0.049 ** ± 0.044
	Suppression (%)	—	19.3 ± 9.1	17.8 ± 13.5	77.8 ** ± 19.7
**Sensitizer**	**Test groups**	**8,8′-bieckol**	**PFF-A**	**PFF-B**	**EGCG**
AA	Ear swelling (mm)	0.153 ± 0.011	0.134 * ± 0.021	0.112 ** ± 0.030	0.168 ± 0.008
	Suppression (%)	21.0 ± 5.5	30.5 ± 10.6	42.2 ^#^ ± 15.7	12.9 ± 4.2
TPA	Ear swelling (mm)	0.132 * ± 0.014	0.132 * ± 0.007	0.119 ** ± 0.031	0.167 ± 0.008
	Suppression (%)	31.7 ± 7.4	31.7 ± 3.8	38.4 ^#^ ± 16.1	13.8 ± 4.4
OXA	Ear swelling (mm)	0.151 * ± 0.030	0.171 ± 0.019	0.131 ** ± 0.032	0.210 ± 0.001
	Suppression (%)	32.3 ^#^ ± 13.6	23.4 ± 8.6	41.0 * ± 14.5	5.7 ± 0.1

Suppression effects at a dose of 75 nmol/mouse were calculated from the results of quadruplicate experiments (n = 4). Controls were stimulated by sensitizer without phlorotannins. Values are shown as means ± standard deviations. In each sensitizer experiment, the presence of an asterisk in “Ear swelling” indicates that the ear swelling was significantly lower than that of control (*, *p* < 0.05; **, *p* < 0.01). An asterisk in “Suppression” indicates that the suppression ratio was significantly higher than that of EGCG (*, *p* < 0.05; **, *p* < 0.01), and “^#^” indicates that it tended to be higher than that of EGCG (0.5 < *p* < 1.0). Abbreviations: AA, arachidonic acid; TPA, 12-*O*-tetradecanoylphorbol-13-acetate; OXA, oxazolone; PFF, phlorofucofuroeckol; EGCG, epigallocatechin gallate.

**Table 2:** The suppressive effects of phlorotannins on degranulation in RBL-2H3 cells stimulated by antigen-antibody or A23187.

**Stimulant**	**Samples**		**Eckol**	**6,6** **′** **-Bieckol**	**6,8** **′** **-Bieckol**	**8,8** **′** **-Bieckol**
Antigen-antibody	Suppression ratio (%)	10 μM	17.3 ± 0.2	3.8 ± 2.3	5.7 ± 3.9	68.2 ** ± 15.8
		100 μM	76.3 ± 4.6	14.5 ± 4.1	68.2 ± 5.8	99.7 ** ± 11.9
A23187	Suppression ratio (%)	10 μM	NA	NA	4.2 ± 2.0	19.3 ** ± 2.3
		100 μM	11.5 ± 5.1	11.2 ± 1.8	35.5 ± 3.3	82.5 ± 2.2
**Stimulant**	**Samples**		**PFF-A**	**PFF-B**	**EGCG**	
Antigen-antibody	Suppression ratio (%)	10 μM	70.1 ** ± 6.8	NA	21.2 ± 10.4	
		100 μM	127.8 ** ± 5.6	44.9 ± 2.6	75.4 ± 2.1	
A23187	Suppression ratio (%)	10 μM	NA	NA	5.2 ± 0.1	
		100 μM	85.2 ^#^ ± 0.7	34.5 ± 1.2	80.0 ± 2.0	

The suppression effects of phlorotannins were calculated from the results of triplicate experiments (n = 3). Values are shown as means ± standard deviations. In each stimulant experimant, the presence of an asterisk indicates that the suppression ratio was significantly (*p* < 0.01) higher than that of EGCG, and “^#^” indicates that it tended to be higher than that of EGCG (0.5 < *p* < 1.0). “NA” indicates no activity. Abbreviations: PFF, phlorofucofuroeckol; EGCG, epigallocatechin gallate.

**Table 3:** The suppression effects of phlorotannins on chemical mediator release in RBL-2H3 cells stimulated by antigen-antibody.

**Chemical mediator**	**Samples**		**Eckol**	**6,6** **′** **-Bieckol**	**6,8** **′** **-Bieckol**	**8,8** **′** **-Bieckol**
Histamine	Suppression ratio (%)	10 μM	15.6 ± 1.2	NA	NA	58.4 ± 19.5
		100 μM	85.1 ** ± 2.9	33.3 ± 2.8	29.4 ± 9.8	84.4 ** ± 4.5
		200 μM	—	—	—	—
Leukotriene B_4_	Suppression ratio (%)	10 μM	10.3 ± 2.3	NA	12.2 ± 0.1	13.4 ± 0.4
		100 μM	66.6 ± 3.4	NA	57.9 ± 1.6	62.7 ± 4.9
		200 μM	—	19.2 ± 3.0	—	—
Prostaglandin E_2_	Suppression ratio (%)	10 μM	10.3 ± 0.7	4.3 ± 1.6	6.9 ± 5.3	12.7 ± 0.3
		100 μM	60.7 ± 0.9	15.3 ± 2.7	36.6 ± 2.7	63.3 ± 3.4
**Chemical mediator**	**Samples**		**PFF-A**	**PFF-B**	**EGCG**	
Histamine	Suppression ratio (%)	10 μM	23.9 ± 15.4	NA	NA	
		100 μM	94.2 ** ± 2.0	NA	36.1 ± 0.1	
		200 μM	—	37.1 ± 24.7	—	
Leukotriene B_4_	Suppression ratio (%)	10 μM	8.9 ± 0.6	3.4 ± 0.4	12.4 ± 7.1	
		100 μM	61.3 ± 5.1	72.5 ± 2.2	71.7 ± 4.3	
		200 μM	—	—	—	
Prostaglandin E_2_	Suppression ratio (%)	10 μM	14.4 ± 1.8	7.1 ± 2.0	17.4 ± 0.2	
		100 μM	82.9 ± 1.3	41.5 ± 11.2	80.3 ± 3.1	

The suppression effects of phlorotannins were calculated from the results of triplicate experiments (n = 3). Values are shown as means ± standard deviations. In the histamine release assay, an asterisk indicates that the suppression ratio was significantly (*p* < 0.01) higher than that of EGCG. “NA” indicates no activity. “—” indicates without examinations. Abbreviations: PFF, phlorofucofuroeckol; EGCG, epigallocatechin gallate.
